# The apoM/S1P Complex—A Mediator in Kidney Biology and Disease?

**DOI:** 10.3389/fmed.2021.754490

**Published:** 2021-10-14

**Authors:** Line S. Bisgaard, Christina Christoffersen

**Affiliations:** ^1^Department of Clinical Biochemistry, Rigshospitalet, Copenhagen, Denmark; ^2^Department of Biomedical Sciences, University of Copenhagen, Copenhagen, Denmark

**Keywords:** apolipoprotein M, sphingosine-1-phosphate, kidney failure, proteinuria, chronic kidney disease, lipoproteins

## Abstract

Kidney disease affects more than 10% of the population, can be both acute and chronic, and is linked to other diseases such as cardiovascular disease, diabetes, and sepsis. Despite the detrimental consequences for patients, no good treatment options directly targeting the kidney are available. Thus, a better understanding of the pathology and new treatment modalities are required. Accumulating evidence suggests that the apolipoprotein M/sphingosine−1-phosphate (apoM/S1P) axis is a likely drug target, but significant gaps in our knowledge remain. In this review, we present what has so far been elucidated about the role of apoM in normal kidney biology and describe how changes in the apoM/S1P axis are thought to affect the development of kidney disease. ApoM is primarily produced in the liver and kidneys. From the liver, apoM is secreted into circulation, where it is attached to lipoproteins (primarily HDL). Importantly, apoM is a carrier of the bioactive lipid S1P. S1P acts by binding to five different receptors. Together, apoM/S1P plays a role in several biological mechanisms, such as inflammation, endothelial cell permeability, and lipid turnover. In the kidney, apoM is primarily expressed in the proximal tubular cells. S1P can be produced locally in the kidney, and several of the five S1P receptors are present in the kidney. The functional role of kidney-derived apoM as well as plasma-derived apoM is far from elucidated and will be discussed based on both experimental and clinical studies. In summary, the current studies provide evidence that support a role for the apoM/S1P axis in kidney disease; however, additional pre-clinical and clinical studies are needed to reveal the mechanisms and target potential in the treatment of patients.

## Introduction

The etiology leading to the development of kidney disease is diverse, but the disease can be divided into two categories, acute or chronic kidney disease, which are both characterized by a decrease in kidney function. Chronic kidney disease is estimated to affect more than 10% of the population worldwide, and up to 5% develop acute kidney injury during hospitalization. Unless resolved, the disease will result in a progressive loss of kidney function and, eventually, the need for dialysis or kidney transplantation. Kidney disease is also linked to other diseases, such as cardiovascular diseases, diabetes, and sepsis. Despite the detrimental consequences for patients, no good treatment options directly targeting kidney function are yet available. Thus, a better understanding of the pathology and new treatment modalities are required.

Apolipoproteins (apo)s are found associated with lipoproteins and assist with the structural stability of the particles as well as the formation, secretion, and uptake of lipoproteins via receptor-mediated pathways. Furthermore, some apos are also involved in inflammation and oxidation and function as chaperones of vitamins or bioactive lipids among others (1–5). So far, apoA through R have been discovered. ApoM was discovered in 1999 by Xu and Dahlbäck, and it was later revealed that apoM acts as the principal carrier of S1P in plasma (5, 6). Since its discovery, apoM has been suggested to be involved in mechanisms such as lipoprotein turnover, especially LDL, and to have anti-inflammatory and atheroprotective effects in part via an improved HDL cholesterol efflux capacity and anti-oxidative effect (7–10). Also, the apoM/S1P complex is involved in maintaining a normal endothelial cell barrier and participates in the regulation of triglyceride metabolism (5, 11). Thus, mice that do not express apoM have increased vessel permeability, increased amounts of brown adipose tissue, and improved triglyceride turnover (5, 11). In contrast, mice overexpressing apoM have delayed triglyceride turnover (12). In addition, apoM has been linked to diabetes [recently reviewed by Christoffersen (13)] and sepsis (14, 15). In a recent study, it was also shown that low apoM levels are associated with adverse outcomes in patients with heart failure (16). Finally, accumulating evidence points toward a role for apoM in fibrosis formation both in the liver and lungs (17, 18). However, the role of apoM in kidney biology and disease has received much less attention. The increasing knowledge from other studies related to different organs or diseases suggests that apoM might also play a role in the pathology of kidney disease. This will be discussed further in this review.

## ApoM and Lipoproteins

### Regulation of ApoM Expression

ApoM is primarily expressed in the liver and kidneys but has also been detected in the intestine, and recent studies suggest that apoM is also expressed in brain endothelial cells and adipocytes (19–22). The apoM gene is localized within the MHC class III region of the genome containing genes involved in the immune system, and it is therefore speculated that apoM might be regulated by inflammatory stimuli. This link has been supported by a study that showed that inflammation results in decreased apoM expression (23). Furthermore, several cytokines [e.g., Transforming growth factor alpha (TGFα), Transforming growth factor beta (TGFβ), Epidermal growth factor (EGF), and Hepatocyte growth factor (HGF)] downregulate apoM expression in liver cells, with TGFβ having the most pronounced effect (24). In contrast, Interleukin 1 alpha (IL-1α) and tumor necrosis factor alpha (TNFα) did not affect apoM expression in HepG2 cells, whereas platelet-activating factor (PAF) stimulation increased apoM expression (25).

ApoM expression in the liver is also regulated by transcription factors and hormones [reviewed in detail by Ren (26)]. Stimulation of HepG2 cells with propofol, a commonly used anesthetic, resulted in a ~3-fold upregulation of apoM expression. This increase is seen concomitantly with an increase in the transcription factors HNF-1α and FOXA2, suggesting that they are both involved in regulating apoM expression (27, 28). A role for HNF-1α is further supported by the finding that HNF-1α deficient mice have decreased apoM expression (29), while Wolfrum et al. have shown that inactivation of FOXA2 leads to lower apoM levels (30). In contrast, stimulation of the transcription factor LXR with the artificial ligand T0901317 results in decreased apoM expression in the liver both *in vivo* and *in vitro* (31, 32), although the same LXR activation leads to increased apoM expression in the intestine (20).

Leptin and insulin seem to be the two most important hormones in the regulation of apoM expression. In humans with hyperinsulinemia, plasma apoM is reduced by ~10% (33, 34), while experimental models with hyperinsulinemia display a reduction in apoM levels of ~50% (35). In addition, Xu et al. demonstrated that insulin stimulation of HepG2 cells leads to a dose- and time-dependent downregulation of apoM (36). It is plausible that this is mediated by insulin-regulated FOXA2 activation (30, 37). Leptin plays an important role in fat metabolism and appetite regulation, and mice with leptin deficiency develop experimental metabolic syndrome and insulin resistance. These mice also have markedly lower apoM levels compared to controls, which is normalized by leptin replenishment (38). In contrast, leptin stimulation of HepG2 cells *in vitro* decreases apoM expression (39). The reason for this apparent discrepancy between the *in vivo* and *in vitro* roles of leptin is unclear and requires further investigation. However, it could at least to some extent, reflect the fact that leptin is a multifunctional hormone that affects many different metabolic pathways *in vivo*, which indirectly modulates apoM expression.

The current knowledge on the regulation of apoM expression suggests the existence of a rather complex system with interactions between transcription factors and hormones. How the different modulators affect each other needs to be further assessed, as well as the potential differences between liver and kidney-derived apoM. To date, no knowledge is available on the specific regulatory mechanisms of apoM expression in the kidney.

### ApoM and Triglyceride Rich Lipoproteins

ApoM is 25 kDa, a member of the lipocalin protein superfamily, and is characterized by an antiparallel β-barrel structure that forms a binding pocket for small hydrophobic molecules (40, 41). In addition, the first 20 amino acids of the apoM protein form a hydrophobic α-helix containing a signal peptide (41). The signal peptide anchors the apoM protein into the phospholipid layer of lipoproteins (42, 43). In 2011, Christoffersen et al. showed that apoM can bind and act as a transporter of S1P (4, 5). ApoM can also bind retinoic acid and retinol in the hydrophobic binding pocket, but the physiological relevance of this is still unexplored (4).

The liver is the primary source of plasma apoM, while the contribution of plasma apoM from the kidneys and other cell types is small, if present. Importantly, apoM is one of the few apolipoproteins, where the hydrophobic signal peptide is not cleaved off during protein maturation. This means that apoM is not present in its free form in the circulation but is instead associated with lipoproteins via its retained signal peptide (42, 43). Thus, 95% of plasma apoM is associated with HDL, but apoM is also present in LDL, VLDL, and chylomicron particles. The relatively low concentration of apoM in plasma (~0.9 μM) means that only ~5% of the HDL particles carry an apoM molecule, while ~2% of LDL particles carry them (44). ApoM is a promiscuous protein that exchanges between lipoproteins in the blood (7, 45). This contrasts with most apolipoproteins that are normally associated with exclusive lipoprotein class (es). The precise mechanism for the exchange of apoM is not clear, but seems to be driven by the availability of different lipoprotein subtypes (7, 45).

Plasma apoM is positively associated not only with the concentration of cholesterol in HDL particles but also with the cholesterol concentration of LDL particles in humans (34). In mouse studies, overexpression of apoM leads to an increased plasma cholesterol concentration, whereas the absence of apoM results in decreased cholesterol concentration (8). At the same time, LDL receptor knockout mice have increased plasma concentrations of apoM, which is also the case in patients carrying single nucleotide polymorphisms in the LDL receptor or apoB (7, 45). Thus, although only a minor portion of apoM is associated with LDL particles, apoM-containing lipoproteins are dependent on the LDL receptor for clearance. Such a link was supported by data reported by our group, who showed that the turnover of apoM-enriched HDL particles injected into LDL receptor knockout mice is slower than that in WT mice (7). In contrast, apoM-containing VLDL/LDL particles had reduced turnover compared to apoM-free VLDL/LDL particles in LDL-receptor-deficient mice, suggesting that apoM may regulate clearance of VLDL/LDL via pathways other than LDL-receptor-mediated uptake.

To date, the role of apoM in triglyceride-rich lipoprotein metabolism in organs other than the liver has not gained much attention. However, apoM is expressed in the intestine and may play a role in triglyceride and chylomicron processing (12, 20). Furthermore, the kidney expresses other apolipoproteins such as apoB100, an apolipoprotein important for LDL and VLDL formation (46). At this point, it is unknown whether apoM expressed in the kidney is similarly involved in the processing of triglycerides containing lipoproteins from the kidney and requires further investigation.

### ApoM and HDL Particles

Due to the hydrophobic nature of the signal peptide, apoM most likely needs a close association with some form of phospholipid layer to be secreted, but the mechanism for this is still unclear. Studies in HEK293 cells and primary hepatocytes overexpressing apoM showed that the signal peptide retains apoM in the intracellular compartment, while cleavage of the signal peptide results in a higher secretion rate of apoM (47, 48). Furthermore, incubation of HEK293 cells overexpressing apoM with serum or HDL stimulates native apoM that are found localized in HDL particles in the medium (47). A similar mechanism was recently found in a study of brain endothelial cells (21). This suggests that apoM can be secreted from cells to lipoprotein particles already present in the plasma. However, how apoM is transported through different intracellular organelles and via the cell membrane to HDL particles, and whether stimulants other than HDL can mediate the secretion, are currently not clear.

While HDL seems to stimulate the secretion of apoM, conversely, apoM also affects HDL biology. However, the data published so far are somewhat contradictory. In brief, nascent HDL particles are primarily formed in the liver in a process where apoAI, the principal apolipoprotein constituent of HDL particles, forms a scaffold at which phospholipids and minor amounts of cholesterol can be associated. This results in the formation of a sub-population of HDL particles called pre-β-HDL (49), which are then secreted from the liver for further maturation in the circulation. These mature HDL particles differ in apoAI content, particle size, and electrophoretic mobility. A study by Wolfrum et al. showed that downregulation of apoM in the liver results in decreased apoAI levels and a lack of pre-β-HDL formation, while there is an accumulation of large HDL particles (10). A similar pattern was seen in HNF-1α deficient mice. These mice have no apoM expression and large buoyant HDL particles (29, 50). In contrast, a subsequent study by our group did not find any obvious difference in HDL particle size in either apoM knockout or apoM transgenic mice (8). In support of this, Mulya et al. have shown that apoM is not necessary for pre-β-HDL formation but is needed to form larger sized particles (51), while Liu et al. showed that the signal peptide is involved in the formation of larger HDL particles (48). Finally, overexpression of apoM in primary hepatocytes induces the formation of larger apoM-enriched HDL particles (52).

In summary, the mechanisms leading to apoM secretion are still poorly understood, but seem to be at least partially induced by HDL particles. At the same time, the predominant data available suggest that apoM plays a role in the formation of larger HDL particles; however, further studies are needed to clarify the mechanism. Finally, how these observations influence and modulate the release of apoM from the kidney remains unknown.

## S1P Expression, Secretion and Signaling

S1P is synthesized from ceramide using sphingosine as an intermediate molecule. The final step of the synthesis is controlled by one of two sphingosine kinases (SPHK1 and 2), both of which are widely expressed in the body. Most cell types can produce S1P, but the S1P concentration is generally low in tissues except the blood. The plasma concentration of S1P ranges from 0.1 to 0.8 μM (53) and is primarily derived from the red blood cells but also from endothelial cells, thrombocytes, mast cells, macrophages, and thrombocytes (54–56). The secretion of S1P from red blood cells is facilitated mainly by the major facilitator superfamily transporter 2b (Mfsd2b). Plasma S1P levels are reduced by 50% in Mfsd2b knockout mice, while the S1P content in red blood cells is markedly increased (57). In addition, several other transporters, including spinster 2, PLTP, and several ABC transporters, have been shown to mediate the secretion of S1P [extensive reviewed by Thuy (58)].

In plasma, several candidates have been suggested as acceptors of S1P, such as apoM, apoAIV, and albumin. Thus, S1P is primarily associated with apoM (65%), and 30% is associated with albumin (5, 59, 60). A more recent study suggested that apoAIV can act as an S1P carrier in the absence of apoM and albumin (61). A study by Sutter et al. showed that apoM-enriched HDL particles enhance the S1P efflux from erythrocytes, while the efflux was comparable when stimulating the release of S1P by, respectively, HDL from apoM knockout mice and WT mice (62). Similar results were obtained by Christensen et al., showing that HDL is more potent than albumin in stimulating S1P release from erythrocytes, and this is enhanced when HDL contains apoM (63). Notably, apoM expression in tissues also seems to enhance S1P expression and secretion. Thus, overexpression of apoM in the liver leads to a higher S1P concentration in both liver tissue and plasma, without affecting the S1P levels in red blood cells, suggesting that S1P can be secreted with apoM from tissues expressing apoM (52, 64). This is supported by studies in HepG2, HeLA, and Raw264.7 cell lines where overexpression of apoM increased the intracellular content of S1P in all three cell types, while secretion was only induced in HepG2 and HeLa cells (64). Furthermore, introduction of a cleavage site for the signal peptide in apoM does not affect S1P expression in hepatocytes, but the secretion of the apoM/S1P complex is increased in cells expressing apoM with the cleavage site (48). Whether other apoM-independent mechanisms mediate S1P release from tissues, such as hepatocytes, remains undetermined. However, pharmacological inhibition of ceramide synthesis leading to increased S1P levels in hepatocytes results in the accumulation of S1P in the cells, suggesting that apoM is rate-limiting for S1P secretion, at least in hepatocytes (48, 52). However, additional studies are needed to reveal the precise mechanism of apoM-induced S1P expression and secretion.

S1P acts as both an intracellular and an extracellular signaling molecule. While the precise mechanism and signaling pathway of intracellular signaling are still only ambiguously described, extracellular signaling is mediated by binding to one of 5 G protein-coupled S1P receptors [sphingosine-1-phosphate receptor 1–5 (S1PR1-5)]. S1P is involved in multiple functions in the body, such as vascular maturation, lymphocyte trafficking, endothelial barrier functions, cell proliferation, and survival. This complexity is mainly thought to arise from the unique expression pattern of S1PRs in different organs and the Ga subunits they activate. Thus, S1PR1 acts via Gi, S1PR2, and 3 via Gi, Gq, or G12/13, while binding to S1P4–5 activates Gi or G12/13. In general, S1PR1 and 3 are believed to mediate similar responses, S1P2 counteracts S1PR1 signaling, while less is known about S1P4 and 5.

Notably, accumulating data suggest that the cellular response to S1P differs depending on whether S1P is bound to apoM or albumin (65). S1P bound to apoM promote a more sustained effect on barrier function compared to S1P bound to albumin, due to a reduction in S1P1 degradation (66). Further, only apoM bound S1P seems to attenuate the endothelial inflammatory response to tumor necrosis factor α, and while albumin bound S1P promote lymphocyte egress, apoM bound S1P inhibits this process (67, 68). The complexity is further highlighted by a study showing that while physiological concentrations of S1P preserve endothelial function, excess levels of S1P results in a dysfunctional endothelial barrier via activation of S1PR2 (69).

## Kidney Derived ApoM—A Mediator of S1P Sequestering or a Mediator of S1P Signaling in the Kidney?

In 2003, Zhang et al. showed that apoM is highly expressed in proximal tubular epithelial cells in kidneys (19). Despite this, the functional role of apoM in kidney biology remains unclear. However, it is hypothesized that apoM can be secreted from proximal tubular cells to the pre-urine. In the pre-urine, apoM may bind otherwise excreted molecules such as S1P and sequester them from excretion by re-uptake into the proximal tubular cells via binding to the megalin receptor (*Lrp2*). This hypothesis is supported by studies showing that apoM can bind to the megalin receptor (70) and that apoM is present in the urine of mice with proximal tubular-specific megalin receptor deficiency, but not in that of WT mice (62, 70). The importance of megalin as a central mediator of apoM uptake in the proximal tubular cells is further supported by a study showing that lack of either the chloride channel ClC-5 (*Clcn5*) or cystinosin (Ctns), which leads to aberrant megalin receptor function, results in excretion of apoM into the urine (62).

What happens to apoM and the molecules that have been sequestered after re-uptake into the proximal tubular cells is even less studied. Likely, molecules such as S1P, which are sequestered in the pre-urine, are released into the peritubular capillaries and thereby returned into the circulation. Whether apoM follows the same route into the circulation or is degraded upon re-uptake or re-shuffled to the pre-urine is unknown. Megalin deficiency in proximal tubular cells and, thereby, loss of apoM to the urine does not affect plasma apoM levels (62), suggesting that apoM is not secreted to the basolateral site of the proximal tubular cells facing the peritubular capillaries. However, these data are only based on western blotting, and could, thus, also reflect that the amounts secreted are below what is possible to detect with such techniques.

Due to the hydrophobic signal peptide, secretion of apoM from the proximal tubular cells would require that apoM is either intracellularly lipidated or that it associates with an intra- or extracellular soluble carrier protein. Faber et al. reported that apoM in the urine of megalin-deficient mice is found in particles that are larger than apoM alone but smaller than HDL particles and devoid of apoAI (70). However, what this particle consists of remains to be determined. Notably, apoM secreted from the liver is already present in the plasma. Thus, the apoM detected in urine from mice with aberrant megalin function could originate from apoM filtered from plasma via the glomeruli. Further supporting this hypothesis, ApoAI and pre-β HDL can be filtered by glomerular capillaries (71). However, apoM was not found to be associated with pre-β HDL in plasma but instead bound to larger HDL particles that likely do not pass the filtration barrier (8, 51, 72). In addition, apoM-containing particles in the urine from megalin-deficient mice do not contain apoAI, further indicating that apoM detected in urine does not originate from plasma but likely from the proximal tubular cells or other cells in the nephron.

The role of kidney-derived apoM as a scavenger of hydrophobic molecules is the only hypothesis put forward for kidney-derived apoM, but other functions may also exist. The accumulation of lipids in non-adipose tissue can be detrimental. This is also the case for the kidney, where lipid accumulation may lead to kidney injury and disease (73). ApoB, the primary apolipoprotein in LDL particles, is expressed in the kidney by proximal tubular epithelial cells, and Krystanek et al. showed that the kidney is able to produce lipoprotein-like apoB-containing particles with a density similar to that of LDL (46). Importantly, knockout of apoB expression leads to the accumulation of triglycerides in the kidney cortex. As apoM is expressed in the same cells as apoB and play a role in metabolism of intestinal derived apoB- and triglyceride-rich lipoproteins (12), an obvious hypothesis is that apoM plays a role in the formation and/or secretion of these particles. If so, apoM could be an important player in regulating lipid metabolism in the kidney, and downregulation could lead to lipid accumulation in kidney cells. However, this is purely speculative and needs to be addressed.

In line with the elusive biological role of apoM in the kidney, several basic features of apoM expression and regulation are still unknown. Thus, whether apoM is expressed in other cell types in the kidney in addition to the expression in the proximal tubular cells have not yet been determined. One study reported that apoM is expressed by mesangial cells (74), but further studies are needed to confirm this finding and determine the level of expression as well as to evaluate its function. In addition, a systematic analysis of the possible expression of apoM in different kidney cells is required. Moreover, no studies have addressed whether the expression of apoM in the kidney is regulated by the same mechanisms as in the liver. The HNF1α transcription factor is expressed in the proximal tubular epithelial cells, as well as LXR, whereas FOXA2 is not expressed in the adult mouse kidney (75–77). This suggests that the regulation differs, at least to some degree, but this is purely speculative and needs to be investigated.

**Figure 1:**
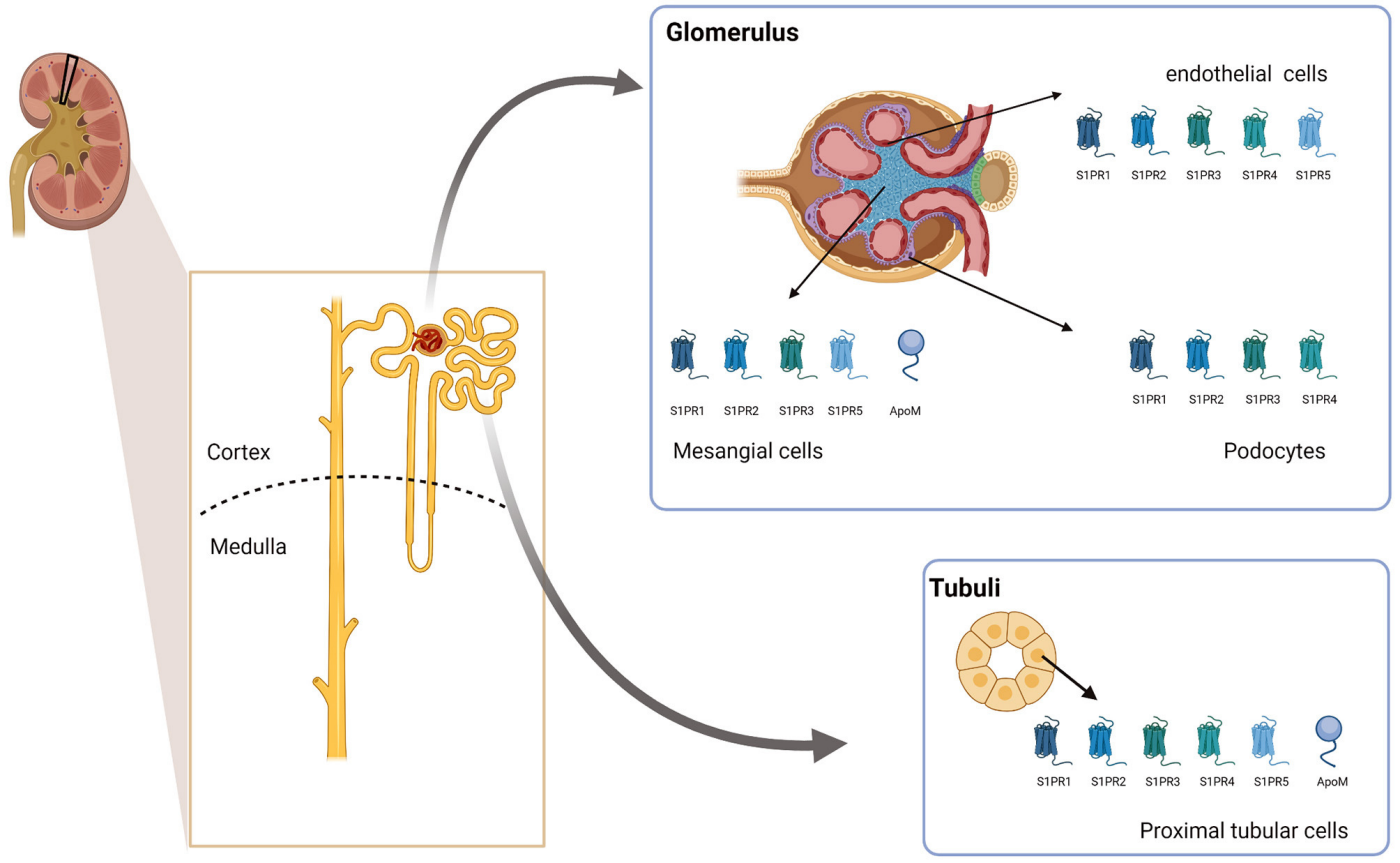
ApoM and S1PR expression in the kidney. The schematic figure summarizes the current knowledge on which cell types in the kidney express apoM and different S1PRs. Created with BioRender.com. ApoM, apolipoprotein M; S1PR1-5, Sphingosine-1-phosphat receptor 1–5.

S1PR1-S1PR4 is expressed in the kidney, with 1 and 3 having the highest expression, followed by receptor 2, while 4 had the lowest expression level. Divergent data exist for receptor 5, with some studies finding it expressed while other studies do not (78, 79). As the kidney is a complex organ with many different cell types, structures, and functions, the location of the expression might be highly relevant for the functional role of S1P. So far, no systematic analysis of the expression pattern of the different receptors in the kidney and their relative expression has been published. However, *in vitro* studies have shown that S1PR1, S1PR2, S1PR3, and S1PR5 are expressed in glomerular mesangial cells (80, 81). All five S1PRs are expressed in glomerular endothelial cells (82) and proximal tubular cells (83, 84), while S1PR1 to S1PR4 are detected in immortalized mouse podocytes (85). Thus, while the expression of apoM seems to be cell-specific, S1PR1-S1PR3 is expressed in both the renal cortex as well as in the inner and outer medulla (summarized in Figure 1). Therefore, it could be speculated that if apoM carrying S1P is secreted locally in the kidney from the proximal tubular cells, it may have local effects in the kidney, even if the secretion is too low to affect the overall plasma concentration of apoM/S1P. However, further studies are required to clarify whether this is biologically relevant. Interestingly, increasing number of scRNAseq data on single cells are available in different databases such as the Kidney Interactive Transcriptomics initiative. These databases can provide new insight into which cell types express which of the S1P receptors as well as apoM and potentially also new biological understandings.

In summary, apoM is expressed in proximal tubular epithelial cells, where it is likely secreted to the pre-urine to sequester small lipophilic molecules such as S1P from excretion. However, the nature of such molecules and their faith, as well as the faith of apoM, remain unknown. Furthermore, it is unclear whether kidney-derived apoM plays a role in other physiological pathways. S1PRs are expressed throughout the kidney. Thus, it could be speculated that sequestered S1P in a complex with apoM has local effects on the kidney.

## Association of ApoM and S1P Levels with Kidney Function in Kidney Disease

The plasma concentration of apoM is affected by different conditions and diseases. Thus, BMI and T2D are inversely associated with apoM levels, while familial hypercholesterolemia with an isolated increase in LDL-C level is associated with an increase in plasma levels of apoM (7, 13). The literature on apoM levels in patients with CKD is heterogeneous and is presented in Table 1. Patients with IgAV-induced nephritis have increased apoM and S1P levels compared to healthy controls but lower levels than those without nephritis (86). These results are supported by an animal study performed in HIGA mice (an experimental model of IgA nephropathy) that found that HIGA mice have increased apoM plasma levels compared to those in control mice (91). In contrast, apoM levels in patients with CKD of mixed etiology are negatively associated with CKD severity (87), while plasma apoM levels in patients with end-stage renal disease are lower than those in controls (88). In patients with primary nephrotic syndrome, plasma apoM is reduced, as well as in patients with hyperlipidemia (90). Furthermore, apoM levels were positively associated with albuminuria in patients with primary nephrotic syndrome (90), while no difference in apoM levels was found when comparing patients with micro-, normo-, and macroalbuminuria in a diabetic cohort (89). The discrepancies found for the association of apoM and kidney function in the different studies may, to some extent, reflect that the underlying etiology for developing CKD is very diverse and that conditions other than kidney disease itself are more important for determining apoM levels. Thus, diabetes is today one of the most common diseases leading to CKD, and patients with T2D have decreased apoM levels, which might at least partially explain the low apoM levels seen in patients with CKD. In line with this, Sorensen et al. found that plasma apoM levels are lower in patients with CKD combined with T2D than in patients with CKD without diabetes (87). Furthermore, induction of mild kidney disease by 5/6-nephrectomy in mice resulted in a somewhat surprising increase in plasma apoM levels. However, the study was conducted in apoE-deficient mice, which are known to have aberrant lipoprotein metabolism and elevated plasma cholesterol levels. Elevated plasma cholesterol levels are as described earlier associated with an increase in apoM levels. Thus, the apoM levels observed in this study may be explained by the cholesterol levels and not the kidney disease, again supporting the idea that circumstances other than kidney disease itself determine apoM levels in patients with CKD (92). This is further supported by the observation that patients with CKD in contrast to the apoE-deficient 5/6-nephrectomized mice normally do not have elevated cholesterol levels (if anything it decreases with decreasing kidney function) and have decreased apoM levels. In addition, streptozotocin-induced type 1 diabetes resulted in increased apoM levels, which were reversed by insulin treatment, while age induced a decrease in apoM levels in mice (93). Together, this highlights the complexity of establishing the underlying cause for a change in plasma apoM levels. Of note, apoM is normally not detectable in the urine, but Svarrer et al. reported that apoM can be detected in urine from pediatric patients with acute kidney injury post heart surgery (94). This is believed to be caused by a direct injury to the proximal tubular cells that are then not able to re-uptake apoM secreted to the urinary site. The study did not measure plasma apoM levels, but it cannot be excluded that pathological changes in the kidney, can induce loss of apoM in the urine and potentially contribute to changes in plasma apoM levels. Further studies are however needed to clarify this.

**Table 1:** Studies of patients or animals with kidney disease and effects on apoM levels.

**Patient group**	**Number of participants**	**Method for apoM assessment**	**Plasma apoM**	**Lipid profile**	**Confounders**	**Albuminuria/proteinuria**	**References**
IgAV	185 (76)	Commercial elisa	**↑**	↑ TC ↑ LDL-C → HDL-C ↓ TG		+	(86)
CKD stage 1-5	409	In-house elisa	**↓**	↓ LDL-C ↓ HDL-C ↑ TG	Diabetes CVD	NA	(87)
ESRD	40 (20)	In-house elisa	**↓**	↓ TC ↓ LDL-C ↓ HDL-C ↑ TG	CVD	NA	(88)
T2D*	90	Commercial elisa	**→**	→ TC → LDL-C → HDL-C → TG	Diabetes CVD	+	(89)
PNS	205 (110)	Commercial elisa	**↓**	↑ TC ↑ LDL-C ↓ HDL-C ↑ TG		+	(90)
Old mice	–	Commercial elisa	**↓**	NA	–	NA	(18)
HIGA mice	–	Western blot	**↑**	NA	–	+	(91)
5/6 NX, mice	–	In-house elisa	**↑**	↑ TC	–	NA	(92)

*ESRD, end-stage renal disease; IgAV, immunoglobulin A–associated vasculitis; CKD, chronic kidney disease; T2D, type 2 diabetes; PNS, primary nephrotic syndrome; 5/6 Nx, 5/6 nephrectomy; TC, total cholesterol; C, cholesterol; LDL, low-density lipoprotein; HDL, high-density lipoprotein; TG, triglycerides. Plasma apoM is increased (↑), decreased (↓) or unchanged (→) compared to control groups. Number of participants are reported as total participants with the number of included healthy controls shown in ()*.

Only a few studies have explored the association between S1P levels and kidney function in patients with CKD. One study has shown that plasma S1P levels in patients with type 2 diabetes decrease concomitantly with kidney function (89). In addition, S1P levels in HDL particles show a tendency to decrease with decreasing kidney function in patients with CKD (87), while S1P plasma levels in pediatric patients with CKD are higher than those in controls (95, 96). In streptozotocin-induced diabetic mice, plasma S1P levels are elevated globally (93) whereas (4 days) diabetic rats have elevated S1P levels locally in glomerular cells (97). Furthermore, berberine treatment of diabetic mice leads to less kidney injury, which is associated with decreased S1P signaling (98). Interestingly, patients with systemic lupus erythematosus had decreased S1P levels compared to controls, but the decrease did not correlate with the plasma albumin levels, suggesting that the dysregulation of S1P is mediated via changes in the apoM/HDL-bound S1P fraction and not the albumin fraction (99). In line with this, S1P decreased more in the HDL fraction than in the albumin fraction in the earlier stages of CKD (87).

Changes in plasma S1P levels could be a result of excess loss via the urine. In support of this idea, results from a study in patients with T2D showed an inverse association between urinary albumin excretion and plasma S1P levels (89). In another study, albuminuria in patients with IgA nephropathy was found to have a positive correlation with both serum S1P and urinary S1P levels (100).

Altogether, the data available points toward a complex biology where the association of both apoM and S1P with kidney disease varies, likely depending on the underlying etiology of the disease. This highlights the complexity of kidney diseases in general and the possible role of apoM and S1P in the development of kidney disease and needs to be included in the interpretation of apoM and/or S1P as biomarkers for kidney disease in future studies. Further, measuring the total plasma S1P might not provide a full picture. Instead, more focus should be given on measuring S1P in albumin and apoM/HDL fractions, respectively.

## The ApoM/S1P Axis—A Mediator of Kidney Disease?

While the association between S1P and kidney function has been poorly studied, the mechanistic role of S1P signaling in kidney disease has been extensively addressed in animal models, recently reviewed in detail by Drexler et al. (65). Thus, in the present review, only a brief summary of the overall trends is provided. Solid evidence points toward a protective effect of S1P signaling in acute kidney disease. In experimental models of ischemia reperfusion (IR) injury, both S1P treatment, an agonist for S1PR in general (FTY720), or specifically, S1PR1 (SEW2871), reduces kidney injury (78, 84, 101–103). Furthermore, SEW2871 stimulation of proximal tubular epithelial cells *in vitro* protected the cells from IR-induced apoptosis, while mice with a proximal tubular specific knockout of S1PR1 have a greater decline in kidney function after IR injury (83, 84). Similarly, mice with a proximal tubular specific knockout of S1PR1 develop more kidney injury after cisplatin injury, which could not be rescued by FTY720 treatment, indicating that local S1P stimuli of the proximal tubular cells are essential for the protective effect (104). In addition, S1P signaling in endothelial cells appears to be important in the development of acute kidney disease as selective deletion of S1PR1 in endothelial cells exacerbates kidney injury upon IR (105).

Only a few studies have examined the role of receptors other than S1PR1, but it has been suggested that S1PR3 signaling aggravates kidney injury after IR (106). In addition, in contrast to the findings for S1PR1, inhibition of S1PR2 signaling is protective against IR injury (107).

Similar to what has been found for acute kidney disease, the literature in general supports a protective role for S1P signaling in chronic kidney disease, although fewer studies are available. In a chronic model of IR injury, mice with an endothelial knockout of S1PR1 develop a more severe injury than WT mice (108). In addition, FTY720 treatment alleviates kidney injury in a rat model of polycystic kidney disease (109), and in a model of early stage diabetic nephropathy, both FTY720 and an S1PR1 specific agonist attenuates kidney injury (85). In contrast, suppression of S1PR2 signaling protects against experimental diabetic nephropathy (110). The protective role of S1PR1 signaling seems to be relevant both in the prevention of injury and in the recovery phase. Thus, knockout of endothelial S1PR1 expression before injury leads to exacerbation of kidney injury after IR, while knockout after IR results in increased tissue fibrosis and impaired healing (108). In humans, sphingosine-1-phosphate lyase (SGPL1) deficiency has been linked to nephrotic syndrome. SGLP1 mediates the degradation of S1P, meaning that deficiency results in accumulation of S1P and increased plasma levels, which further supports a causal role for S1P in kidney disease (111–113).

Little is known about the role of apoM in kidney diseases. However, accumulating data suggest that apoM is not only affected by kidney disease, but also has a causal role. In a recent study by Ding et al., reduced apoM levels resulted in increased kidney injury (18). Similarly, adenovirus-induced overexpression of apoM results in less kidney injury in HIGA mice and reduced expression of classical markers of fibrosis (incl. TGFβ and FN-1), while siRNA-induced apoM deletion results in accelerated injury compared to control animals (91). The mechanisms underlying the effect of apoM on the kidney are not clear; however, S1P bound to albumin results in increased proliferation of mesangial cells and increased expression of pro-fibrotic genes in the proximal epithelial cells; these effects were not observed when the cells were stimulated with S1P bound to apoM. To this point, only one study has addressed the role of kidney-derived apoM in kidney disease. This study suggests that silencing of apoM expression in mesangial cells leads to mitochondrial damage and apoptosis (74). However, apoM is mainly expressed in the proximal tubular cells. Therefore, it would be of great importance to unravel whether apoM derived from these cells has any role locally in the kidney during kidney injury.

Taken together, these data suggest that local S1P signaling in the kidney is of high importance for protecting the kidney against injury. What role both plasma and kidney-derived apoM play in connection to this, however, still needs to be addressed in future studies. Though highly speculative the data so far available could suggest that the apoM/S1P-complex act in two separate ways in the kidney—(1) being a systemic effect of circulating HDL-apoM/S1P or albumin-apoM/S1P on endothelial cells in the kidney and (2) a direct effect of kidney derived apoM on tubular cells. This is important to keep in mind as these two pathways might not have the same effect and regulation, which could influence how potential drug candidates targeting the apoM/S1P axis act in different diseases.

## Conclusion

As this review suggests and highlights, significant gaps in our knowledge still need to be elucidated before we understand the role and function of kidney-produced apoM and S1P, but also whether the plasma lipoproteins containing apoM and S1P play a role in biological processes that affect kidney function and pathologies. Drugs targeting the S1P-receptor pathways are evolving as well as apoM-modulating drugs with a focus on cardiovascular diseases and inflammation; however, only a few of these studies have also reported data that are relevant to kidney biology. For example, pharmacological targeting of S1PR1 can improve renal microcirculation during sepsis in mice (114). Acute kidney failure or organ failure is a well-known consequence of severe sepsis. Likewise, treating animals with an artificial apoM-FC component can attenuate hypertension and IR injury (115). Whether any of these drugs will reach a level of clinical use will be very interesting to follow the years ahead. Hopefully, and in parallel, the community will gain further information from upcoming pre-clinical studies on potential side effects of such drugs related to, for example, lipid accumulation or compromised inflammatory response, both harmful to the kidney.

## Author Contributions

LB and CC designed and wrote this manuscript. All authors contributed to the article and approved the submitted version.

## Funding

This work was funded by the Danish Diabetes Academy, the Novo Nordisk Foundation and Augustinus Foundation.

## Conflict of Interest

The authors declare that the research was conducted in the absence of any commercial or financial relationships that could be construed as a potential conflict of interest.

## Publisher's Note

All claims expressed in this article are solely those of the authors and do not necessarily represent those of their affiliated organizations, or those of the publisher, the editors and the reviewers. Any product that may be evaluated in this article, or claim that may be made by its manufacturer, is not guaranteed or endorsed by the publisher.

## References

[1] Vuilleumier N Dayer J Von E Roux-Lombard P. Pro- or anti-inflammatory role of apolipoprotein A-1 in high-density lipoproteins? Swiss Med Weekly. (2013) 143:W13781. 10.4414/smw.2013.13781 23740387

[2] Berbee JF Havekes LM Rensen PC. Apolipoproteins modulate the inflammatory response to lipopolysaccharide. J Endotoxin Res. (2005) 11:97–103. 10.1177/09680519050110020501 15949136

[3] Nicod N Parker RS. Vitamin E secretion by Caco-2 monolayers to APOA1, but Not to HDL, is vitamer selective. J Nutr. (2013) 143:1565–72. 10.3945/jn.113.176834 23946344 PMC3771812

[4] Ahnström J Faber K Axler O Dahlbäck B. Hydrophobic ligand binding properties of the human lipocalin apolipoprotein M. J Lipid Res. (2007) 48:1754–62. 10.1194/jlr.M700103-JLR200 17525477

[5] Christoffersen C Obinata H Kumaraswamy SB Galvani S Ahnstrom J Sevvana M. Endothelium-protective sphingosine-1-phosphate provided by HDL-associated apolipoprotein M. Proc Natl Acad Sci USA. (2011) 108:9613–8. 10.1073/pnas.1103187108 21606363 PMC3111292

[6] Xu N Dahlback B. A novel human apolipoprotein (apoM). J Biol Chem. (1999) 274:31286–90. 10.1074/jbc.274.44.31286 10531326

[7] Christoffersen C Benn M Christensen PM Gordts PL Roebroek AJ Frikke-Schmidt R. The plasma concentration of HDL-associated apoM is influenced by LDL receptor-mediated clearance of apoB-containing particles. J Lipid Res. (2012) 53:2198–204. 10.1194/jlr.P023697 22826357 PMC3435552

[8] Christoffersen C Jauhiainen M Moser M Porse B Ehnholm C Boesl M. Effect of apolipoprotein M on high density lipoprotein metabolism and atherosclerosis in low density lipoprotein receptor knock-out mice. J Biol Chem. (2008) 283:1839–47. 10.1074/jbc.M704576200 18006500

[9] Elsoe S Ahnstrom J Christoffersen C Hoofnagle AN Plomgaard P Heinecke JW. Apolipoprotein M binds oxidized phospholipids and increases the antioxidant effect of HDL. Atherosclerosis. (2012) 221:91–7. 10.1016/j.atherosclerosis.2011.11.031 22204862

[10] Wolfrum C Poy MN Stoffel M. Apolipoprotein M is required for prebeta-HDL formation and cholesterol efflux to HDL and protects against atherosclerosis. Nat Med. (2005) 11:418–22. 10.1038/nm1211 15793583

[11] Christoffersen C Federspiel CK Borup A Christensen PM Madsen AN Heine M. The apolipoprotein M/S1P axis controls triglyceride metabolism and brown fat activity. Cell Reports. (2018) 22:175–88. 10.1016/j.celrep.2017.12.029 29298420

[12] Hajny S Borup A Elsoe S Christoffersen C. Increased plasma apoM levels impair triglyceride turnover in mice. Biochim Biophys Acta Mol Cell Biol Lipids. (2021) 1866:158969. 10.1016/j.bbalip.2021.158969 34051379

[13] Christoffersen C. Apolipoprotein M-A marker or an active player in Type II diabetes? Front Endocrinol. (2021) 12:665393. 10.3389/fendo.2021.665393 34093440 PMC8176018

[14] Kurano M Tsuneyama K Morimoto Y Shimizu T Jona M Kassai H. Apolipoprotein M protects lipopolysaccharide-treated mice from death and organ injury. Thromb Haemost. (2018) 118:1021–35. 10.1055/s-0038-1641750 29669385

[15] Frej C Linder A Happonen KE Taylor FB Lupu F Dahlback B. Sphingosine 1-phosphate and its carrier apolipoprotein M in human sepsis and in Escherichia coli sepsis in baboons. J Cell Mol Med. (2016) 20:1170–81. 10.1111/jcmm.12831 26990127 PMC4882985

[16] Chirinos JA Zhao L Jia Y Frej C Adamo L Mann D. Reduced apolipoprotein m and adverse outcomes across the spectrum of human heart failure. Circulation. (2020) 141:1463–76. 10.1161/CIRCULATIONAHA.119.045323 32237898 PMC7200273

[17] Ding BS Liu CH Sun Y Chen Y Swendeman SL Jung B. HDL activation of endothelial sphingosine-1-phosphate receptor-1 (S1P1) promotes regeneration and suppresses fibrosis in the liver. JCI Insight. (2016) 1:e87058. 10.1172/jci.insight.87058 28018969 PMC5161208

[18] Ding BS Yang D Swendeman SL Christoffersen C Nielsen LB Friedman SL. Aging suppresses sphingosine-1-phosphate chaperone ApoM in circulation resulting in maladaptive organ repair. Dev Cell. (2020) 53:677–90 e4. 10.1016/j.devcel.2020.05.024 32544390 PMC7607448

[19] Zhang XY Dong X Zheng L Luo GH Liu YH Ekstrom U. Specific tissue expression and cellular localization of human apolipoprotein M as determined by in situ hybridization. Acta Histochem. (2003) 105:67–72. 10.1078/0065-1281-00687 12666989

[20] Calayir E Becker TM Kratzer A Ebner B Panzenbock U Stefujl J. LXR-agonists regulate ApoM expression differentially in liver and intestine. Curr Pharm Biotechnol. (2008) 9:516–21. 10.2174/138920108786786376 19075690

[21] Kober AC Manavalan APC Tam-Amersdorfer C Holmer A Saeed A Fanaee-Danesh E. Implications of cerebrovascular ATP-binding cassette transporter G1 (ABCG1) and apolipoprotein M in cholesterol transport at the blood-brain barrier. Biochim Biophys Acta Mol Cell Biol Lipids. (2017) 1862:573–88. 10.1016/j.bbalip.2017.03.003 28315462

[22] Sramkova V Berend S Siklova M Caspar-Bauguil S Carayol J Bonnel S. Apolipoprotein M: a novel adipokine decreasing with obesity and upregulated by calorie restriction. Am J Clin Nutr. (2019) 109:1499–510. 10.1093/ajcn/nqy331 30869115

[23] Feingold KR Shigenaga JK Chui LG Moser A Khovidhunkit W Grunfeld C. Infection and inflammation decrease apolipoprotein M expression. Atherosclerosis. (2008) 199:19–26. 10.1016/j.atherosclerosis.2007.10.007 18054359

[24] Xu N Hurtig M Zhang XY Ye Q Nilsson-Ehle P. Transforming growth factor-beta down-regulates apolipoprotein M in HepG2 cells. Biochim Biophys Acta. (2004) 1683:33–7. 10.1016/j.bbalip.2004.04.001 15238217

[25] Xu N Zhang XY Dong X Ekstrom U Ye Q Nilsson-Ehle P. Effects of platelet-activating factor, tumor necrosis factor, and interleukin-1alpha on the expression of apolipoprotein M in HepG2 cells. Biochem Biophys Res Commun. (2002) 292:944–50. 10.1006/bbrc.2002.6755 11944906

[26] Ren K Tang ZL Jiang Y Tan YM Yi GH. Apolipoprotein M. Clin Chim Acta. (2015) 446:21–9. 10.1016/j.cca.2015.03.038 25858547

[27] Ma X Hu YW Zhao ZL Zheng L Qiu YR Huang JL. Anti-inflammatory effects of propofol are mediated by apolipoprotein M in a hepatocyte nuclear factor-1alpha-dependent manner. Arch Biochem Biophys. (2013) 533:1–10. 10.1016/j.abb.2013.03.002 23500137

[28] Ma X Zhao JY Zhao ZL Ye J Li SF Fang HH. Propofol attenuates lipopolysaccharide-induced monocyte chemoattractant protein-1 production through enhancing apom and foxa2 expression in HepG2 cells. Inflammation. (2015) 38:1329–36. 10.1007/s10753-014-0104-y 25586482

[29] Richter S Shih DQ Pearson ER Wolfrum C Fajans SS Hattersley AT. Regulation of apolipoprotein M gene expression by MODY3 gene hepatocyte nuclear factor-1 : haploinsufficiency is associated with reduced serum apolipoprotein M levels. Diabetes. (2003) 52:2989–95. 10.2337/diabetes.52.12.2989 14633861

[30] Wolfrum C Howell JJ Ndungo E Stoffel M. Foxa2 activity increases plasma high density lipoprotein levels by regulating apolipoprotein M. J Biol Chem. (2008) 283:16940–9. 10.1074/jbc.M801930200 18381283

[31] Zhang X Zhu Z Luo G Zheng L Nilsson-Ehle P Xu N. Liver X receptor agonist downregulates hepatic apoM expression in vivo and in vitro. Bioc Bio Res Commun. (2008) 371:114–7. 10.1016/j.bbrc.2008.04.017 18413148

[32] Di D Wang Z Liu Y Luo G Shi Y Berggren-Söderlund M. ABCA1 upregulating apolipoproein M expression mediates via the RXR/LXR pathway in HepG2 cells. Bioc Bio Res Commun. (2012) 421:152–6. 10.1016/j.bbrc.2012.04.022 22516753

[33] Zhang P-H Gao J-L Pu C Feng G Wang L-Z Huang L-Z. A single-nucleotide polymorphism C-724 /del in the proter region of the apolipoprotein M gene is associated with type 2 diabetes mellitus. Lipids Health Dis. (2016) 15:142. 10.1186/s12944-016-0307-3 27576735 PMC5006532

[34] Plomgaard P Dullaart RP de Vries R Groen AK Dahlback B Nielsen LB. Apolipoprotein M predicts pre-beta-HDL formation: studies in type 2 diabetic and nondiabetic subjects. J Intern Med. (2009) 266:258–67. 10.1111/j.1365-2796.2009.02095.x 19457058

[35] Zhang X Jiang B Luo G Nilsson-Ehle P Xu N. Hyperglycemia down-regulates apolipoprotein M expression in vivo and in vitro. Biochim Biophys Acta. (2007) 1771:879–82. 10.1016/j.bbalip.2007.04.020 17556016

[36] Xu N Ahrén B Jiang J Nilsson-Ehle P. Down-regulation of apolipoprotein M expression is mediated by phosphatidylinositol 3-kinase in HepG2 cells. Bioc Bio Acta. (2006) 1761:256–60. 10.1016/j.bbalip.2006.02.002 16542871

[37] Wolfrum C Besser D Luca E Stoffel M. Insulin regulates the activity of forkhead transcription factor Hnf-3 /Foxa-2 by Akt-mediated phosphorylation and nuclear/cytosolic localization. Proc Natl Acad Sci USA. (2003) 100:11624–9. 10.1073/pnas.1931483100 14500912 PMC208808

[38] Xu N Nilsson-Ehle P Hurtig M Ahrén B. Both leptin and leptin-receptor are essential for apolipoprotein M expression in vivo. Bioc Bio Res Commun. (2004) 321:916–21. 10.1016/j.bbrc.2004.06.180 15358114

[39] Luo G Hurtig M Zhang X Nilsson-Ehle P Xu N. Leptin inhibits apolipoprotein M transcription and secretion in human hepatoma cell line, HepG2 cells. Bioc Bio Acta. (2005) 1734:198–202. 10.1016/j.bbalip.2005.02.005 15904876

[40] Duan J Dahlback B Villoutreix BO. Proposed lipocalin fold for apolipoprotein M based on bioinformatics and site-directed mutagenesis. FEBS Lett. (2001) 499:127–32. 10.1016/S0014-5793(01)02544-3 11418126

[41] Sevvana M Ahnstrom J Egerer-Sieber C Lange HA Dahlback B Muller YA. Serendipitous fatty acid binding reveals the structural determinants for ligand recognition in apolipoprotein M. J Mol Biol. (2009) 393:920–36. 10.1016/j.jmb.2009.08.071 19733574

[42] Christoffersen C Ahnström J Axler O Christensen EI Dahlbäck B Nielsen LB. The signal peptide anchors apolipoprotein M in plasma lipoproteins and prevents rapid clearance of apolipoprotein M from plasma. J Bio Chem. (2008) 283:18765–72. 10.1074/jbc.M800695200 18460466

[43] Axler O Ahnström J Dahlbäck B. Apolipoprotein M associates to lipoproteins through its retained signal peptide. FEBS Letters. (2008) 582:826–8. 10.1016/j.febslet.2008.02.007 18279674

[44] Christoffersen C Nielsen LB Axler O Andersson A Johnsen AH Dahlback B. Isolation and characterization of human apolipoprotein M-containing lipoproteins. J Lipid Res. (2006) 47:1833–43. 10.1194/jlr.M600055-JLR200 16682745

[45] Christoffersen C Pedersen TX Gordts PLSM Roebroek AJM DahlbäCk BR Nielsen LB. Opposing effects of apolipoprotein M on catabolism of apolipoprotein B–containing lipoproteins and atherosclerosis. Circ Res. (2010) 106:1624–34. 10.1161/CIRCRESAHA.109.211086 20360257

[46] Krzystanek M Pedersen TX Bartels ED Kjæhr J Straarup EM Nielsen LB. Expression of apolipoprotein b in the kidney attenuates renal lipid accumulation^*^. J Bio Chem. (2010) 285:10583–90. 10.1074/jbc.M109.078006 20103594 PMC2856266

[47] Ahnstrom J Axler O Dahlback B. HDL stimulates apoM secretion. Protein Pept Lett. (2010) 17:1285–9. 10.2174/092986610792231410 20518736

[48] Liu M Allegood J Zhu X Seo J Gebre AK Boudyguina E. Uncleaved ApoM signal peptide is required for formation of large ApoM/Sphingosine 1-Phosphate (S1P)-enriched HDL particles. J Bio Chem. (2015) 290:7861–70. 10.1074/jbc.M114.631101 25627684 PMC4367285

[49] Wroblewska M. The origin and metabolism of a nascent pre-beta high density lipoprotein involved in cellular cholesterol efflux. Acta Biochim Pol. (2011) 58:275–85. 10.18388/abp.2011_2237 21750785

[50] Shih DQ Bussen M Sehayek E Ananthanarayanan M Shneider BL Suchy FJ. Hepatocyte nuclear factor-1alpha is an essential regulator of bile acid and plasma cholesterol metabolism. Nat Genet. (2001) 27:375–82. 10.1038/86871 11279518

[51] Mulya A Seo J Brown AL Gebre AK Boudyguina E Shelness GS. Apolipoprotein M expression increases the size of nascent pre beta HDL formed by ATP binding cassette transporter A1. J Lipid Res. (2010) 51:514–24. 10.1194/jlr.M002162 19767535 PMC2817581

[52] Liu M Seo J Allegood J Bi X Zhu X Boudyguina E. Hepatic apolipoprotein M (ApoM) overexpression stimulates formation of larger ApoM/Sphingosine 1-Phosphate-enriched plasma high density lipoprotein. J Bio Chem. (2014) 289:2801–14. 10.1074/jbc.M113.499913 24318881 PMC3908412

[53] Kharel Y Huang T Salamon A Harris TE Santos WL Lynch KR. Mechanism of sphingosine 1-phosphate clearance from blood. Biochem J. (2020) 477:925–35. 10.1042/BCJ20190730 32065229 PMC7059866

[54] Ksiazek M Chacińska M Chabowski A Baranowski M. Sources, metabolism, and regulation of circulating sphingosine-1-phosphate. J Lipid Res. (2015) 56:1271–81. 10.1194/jlr.R059543 26014962 PMC4479332

[55] Venkataraman K Lee Y-M Michaud J Thangada S Ai Y Bonkovsky HL. Vascular endothelium as a contributor of plasma sphingosine 1-phosphate. Circ Res. (2008) 102:669–76. 10.1161/CIRCRESAHA.107.165845 18258856 PMC2659392

[56] Hänel P Andréani P Gräler MH. Erythrocytes store and release sphingosine 1-phosphate in blood. FASEB J. (2007) 21:1202–9. 10.1096/fj.06-7433com 17215483

[57] Vu TM Ishizu A-N Foo JC Toh XR Zhang F Whee DM. Mfsd2b is essential for the sphingosine-1-phosphate export in erythrocytes and platelets. Nature. (2017) 550:524–8. 10.1038/nature24053 29045386

[58] Thuy AV Reimann C-M Hemdan NYA Gräler MH. Sphingosine 1-phosphate in blood: function, metabolism, and fate. Cell Physiol Bio. (2014) 34:158–71. 10.1159/000362992 24977489

[59] Aoki S Yatomi Y Ohta M Osada M Kazama F Satoh K. Sphingosine 1-phosphate-related metabolism in the blood vessel. J Biochem. (2005) 138:47–55. 10.1093/jb/mvi100 16046448

[60] Argraves KM Argraves WS. HDL serves as a S1P signaling platform mediating a multitude of cardiovascular effects. J Lipid Res. (2007) 48:2325–33. 10.1194/jlr.R700011-JLR200 17698855

[61] Obinata H Kuo A Wada Y Swendeman S Liu CH Blaho VA. Identification of ApoA4 as a sphingosine 1-phosphate chaperone in ApoM- and albumin-deficient mice. J Lipid Res. (2019) 60:1912–21. 10.1194/jlr.RA119000277 31462513 PMC6824498

[62] Sutter I Park R Othman A Rohrer L Hornemann T Stoffel M. Apolipoprotein M modulates erythrocyte efflux and tubular reabsorption of sphingosine-1-phosphate. J Lipid Res. (2014) 55:1730–7. 10.1194/jlr.M050021 24950692 PMC4109767

[63] Christensen PM Bosteen MH Hajny S Nielsen LB Christoffersen C. Apolipoprotein M mediates sphingosine-1-phosphate efflux from erythrocytes. Sci Rep. (2017) 7:14983. 10.1038/s41598-017-15043-y 29118354 PMC5678177

[64] Kurano M Tsukamoto K Ohkawa R Hara M Iino J Kageyama Y. Liver involvement in sphingosine 1-phosphate dynamism revealed by adenoviral hepatic overexpression of apolipoprotein M. Atherosclerosis. (2013) 229:102–9. 10.1016/j.atherosclerosis.2013.04.024 23664237

[65] Drexler Y Molina J Mitrofanova A Fornoni A Merscher S. Sphingosine-1-phosphate metabolism and signaling in kidney diseases. J Am Soc Nephrol. (2021) 32:9–31. 10.1681/ASN.2020050697 33376112 PMC7894665

[66] Wilkerson BA Grass GD Wing SB Argraves WS Argraves KM. Sphingosine 1-Phosphate (S1P) carrier-dependent regulation of endothelial barrier. J Bio Chem. (2012) 287:44645–53. 10.1074/jbc.M112.423426 23135269 PMC3531779

[67] Galvani S Sanson M Blaho VA Swendeman SL Obinata H Conger H. HDL-bound sphingosine 1-phosphate acts as a biased agonist for the endothelial cell receptor S1P1 to limit vascular inflammation. Sci Signal. (2015) 8:ra79. 10.1126/scisignal.aaa2581 26268607 PMC4768813

[68] Blaho VA Galvani S Engelbrecht E Liu C Swendeman SL Kono M. HDL-bound sphingosine-1-phosphate restrains lymphopoiesis and neuroinflammation. Nature. (2015) 523:342–6. 10.1038/nature14462 26053123 PMC4506268

[69] Li Q Chen B Zeng C Fan A Yuan Y Guo X. Differential activation of receptors and signal pathways upon stimulation by different doses of sphingosine-1-phosphate in endothelial cells. Exp Physiol. (2015) 100:95–107. 10.1113/expphysiol.2014.082149 25557733

[70] Faber K Hvidberg V Moestrup SK DahlbäCk BR Nielsen LB. Megalin is a receptor for apolipoprotein m, and kidney-specific megalin-deficiency confers urinary excretion of apolipoprotein M. Mol Endocrinol. (2006) 20:212–8. 10.1210/me.2005-0209 16099815

[71] Yang H Fogo AB Kon V. Kidneys. Curr Opin Nephrol Hypert. (2016) 25:174–9. 10.1097/MNH.0000000000000217 27008596 PMC4899840

[72] Miyazaki O Ogihara J Fukamachi I Kasumi T. Evidence for the presence of lipid-free monomolecular apolipoprotein A-1 in plasma. J Lipid Res. (2014) 55:214–25. 10.1194/jlr.M041038 24304668 PMC3886660

[73] Gai Z Wang T Visentin M Kullak-Ublick G Fu X Wang Z. Lipid accumulation and chronic kidney disease. Nutrients. (2019) 11:722. 10.3390/nu11040722 30925738 PMC6520701

[74] Pei W Wu Y Zhang X Lv K Zhang Y Li Z. Deletion of ApoM gene induces apoptosis in mouse kidney via mitochondrial and endoplasmic reticulum stress pathways. Biochem Biophys Res Commun. (2018) 505:891–7. 10.1016/j.bbrc.2018.09.162 30301532

[75] Yonezawa S Abe M Kawasaki Y Natori Y Sugiyama A. Each liver X receptor (LXR) type has a different purpose in different situations. Biochem Biophys Res Commun. (2019) 508:92–6. 10.1016/j.bbrc.2018.11.076 30471864

[76] Besnard V Wert SE Hull WM Whitsett JA. Immunohistochemical localization of Foxa1 and Foxa2 in mouse embryos and adult tissues. Gene Expr Patterns. (2004) 5:193–208. 10.1016/j.modgep.2004.08.006 15567715

[77] Cheret C Doyen A Yaniv M Pontoglio M. Hepatocyte nuclear factor 1 α controls renal expression of the npt1-npt4 anionic transporter locus. J Mol Biol. (2002) 322:929–41. 10.1016/S0022-2836(02)00816-1 12367519

[78] Awad AS Ye H Huang L Li L Foss FW Jr. Selective sphingosine 1-phosphate 1 receptor activation reduces ischemia-reperfusion injury in mouse kidney. Am J Physiol Renal Physiol. (2006) 290:F1516–24. 10.1152/ajprenal.00311.2005 16403835

[79] Park SW Kim M Chen SW Brown KM D'Agati VD Lee HT. Sphinganine-1-phosphate protects kidney and liver after hepatic ischemia and reperfusion in mice through S1P1 receptor activation. Lab Invest. (2010) 90:1209–24. 10.1038/labinvest.2010.102 20458275 PMC3007623

[80] Imasawa T Kitamura H Ohkawa R Satoh Y Miyashita A Yatomi Y. Unbalanced expression of sphingosine 1-phosphate receptors in diabetic nephropathy. Exp Toxicol Pathol. (2010) 62:53–60. 10.1016/j.etp.2009.02.068 19261455

[81] Koch A Volzke A Puff B Blankenbach K Meyer Zu Heringdorf D Huwiler A. PPARgamma agonists upregulate sphingosine 1-phosphate (S1P) receptor 1 expression, which in turn reduces S1P-induced [Ca(2+)]i increases in renal mesangial cells. Biochim Biophys Acta. (2013) 1831:1634–43. 10.1016/j.bbalip.2013.07.011 23906789

[82] Sun XJ Wang C Zhang LX Yu F Chen M Zhao MH. Sphingosine-1-phosphate and its receptors in anti-neutrophil cytoplasmic antibody-associated vasculitis. Nephrol Dial Transplant. (2017) 32:1313–22. 10.1093/ndt/gfw427 28206609

[83] Kim M Kim M Park SW Pitson SM Lee HT. Isoflurane protects human kidney proximal tubule cells against necrosis via sphingosine kinase and sphingosine-1-phosphate generation. Am J Nephrol. (2010) 31:353–62. 10.1159/000298339 20234131 PMC2859229

[84] Bajwa A Jo SK Ye H Huang L Dondeti KR Rosin DL. Activation of sphingosine-1-phosphate 1 receptor in the proximal tubule protects against ischemia-reperfusion injury. J Am Soc Nephrol. (2010) 21:955–65. 10.1681/ASN.2009060662 20338995 PMC2900956

[85] Awad AS Rouse MD Khutsishvili K Huang L Bolton WK Lynch KR. Chronic sphingosine 1-phosphate 1 receptor activation attenuates early-stage diabetic nephropathy independent of lymphocytes. Kidney Int. (2011) 79:1090–8. 10.1038/ki.2010.544 21289599 PMC3155206

[86] Wu J He L Bai L Tan L Hu M. Apolipoprotein M serum levels correlate with IgA vasculitis and IgA vasculitis nephritis. Dis Markers. (2019) 2019:1825849. 10.1155/2019/1825849 31885732 PMC6927057

[87] Sorensen IM Bertelsen M Freese E Lindhard K Ullum H Feldt-Rasmussen B. Apolipoprotein M in patients with chronic kidney disease. Atherosclerosis. (2018) 275:304–11. 10.1016/j.atherosclerosis.2018.06.815 29980057

[88] Brinck JW Thomas A Brulhart-Meynet M-C Lauer E Frej C Dahlbäck B. High-density lipoprotein from end-stage renal disease patients exhibits superior cardioprotection and increase in sphingosine-1-phosphate. Eur J Clin Invest. (2018) 48:e12866. 10.1111/eci.12866 29178180

[89] Bekpinar S Yenidunya G Gurdol F Unlucerci Y Aycan-Ustyol E Dinccag N. The effect of nephropathy on plasma sphingosine 1-phosphate concentrations in patients with type 2 diabetes. Clin Biochem. (2015) 48:1264–7. 10.1016/j.clinbiochem.2015.08.001 26255120

[90] He L Wu P Tan L Le B Du W Shen T. Characteristics of lipid metabolism including serum apolipoprotein M levels in patients with primary nephrotic syndrome. Lipids Health Dis. (2017) 16:167. 10.1186/s12944-017-0556-9 28877724 PMC5585964

[91] Kurano M Tsuneyama K Morimoto Y Nishikawa M Yatomi Y. Apolipoprotein M suppresses the phenotypes of IgA nephropathy in hyper-IgA mice. FASEB J. (2019) 33:5181–95. 10.1096/fj.201801748R 30629456

[92] Bosteen MH Madsen Svarrer EM Bisgaard LS Martinussen T Madsen M Nielsen LB. Effects of apolipoprotein M in uremic atherosclerosis. Atherosclerosis. (2017) 265:93–101. 10.1016/j.atherosclerosis.2017.08.005 28866363

[93] Nojiri T Kurano M Tokuhara Y Ohkubo S Hara M Ikeda H. Modulation of sphingosine-1-phosphate and apolipoprotein M levels in the plasma, liver and kidneys in streptozotocin-induced diabetic mice. J Diab Invest. (2014) 5:639–48. 10.1111/jdi.12232 25422763 PMC4234226

[94] Madsen Svarrer EM Andersen HØ Helvind M Slagman MC Navis G Dullaart RP. Urinary apolipoprotein M as a biomarker of acute kidney injury in children undergoing heart surgery. Bio Med. (2016) 10:81–93. 10.2217/bmm.15.105 26642098

[95] Benito S Sánchez-Ortega A Unceta N Jansen JJ Postma G Andrade F. Plasma biomarker discovery for early chronic kidney disease diagnosis based on chemometric approaches using LC-QTOF targeted metabolomics data. J Pharm Biomed Anal. (2018) 149:46–56. 10.1016/j.jpba.2017.10.036 29100030

[96] Benito S Sanchez-Ortega A Unceta N Goicolea MA Barrio RJ. LC-QQQ-MS routine analysis method for new biomarker quantification in plasma aimed at early chronic kidney disease diagnosis. J Pharm Biomed Anal. (2019) 169:82–9. 10.1016/j.jpba.2019.02.042 30844626

[97] Geoffroy K Troncy L Wiernsperger N Lagarde M El Bawab S. Glomerular proliferation during early stages of diabetic nephropathy is associated with local increase of sphingosine-1-phosphate levels. FEBS Lett. (2005) 579:1249–54. 10.1016/j.febslet.2004.12.094 15710421

[98] Lan T Shen X Liu P Liu W Xu S Xie X. Berberine ameliorates renal injury in diabetic C57BL/6 mice: Involvement of suppression of SphK-S1P signaling pathway. Arch Biochem Biophys. (2010) 502:112–20. 10.1016/j.abb.2010.07.012 20646989

[99] Checa A Idborg H Zandian A Sar DG Surowiec I Trygg J. Dysregulations in circulating sphingolipids associate with disease activity indices in female patients with systemic lupus erythematosus: a cross-sectional study. Lupus. (2017) 26:1023–33. 10.1177/0961203316686707 28134039

[100] Su K Zeng P Liang W Luo Z Wang Y Lv X. FTY720 attenuates angiotensin ii-induced podocyte damage via inhibiting inflammatory cytokines. Med Inflamm. (2017) 2017:3701385. 10.1155/2017/3701385 28270699 PMC5320072

[101] Lee S-Y Kim D-H Sung S-A Kim M-G Cho W-Y Kim H-K. Sphingosine-1-phosphate reduces hepatic ischaemia/reperfusion-induced acute kidney injury through attenuation of endothelial injury in mice. Nephrology. (2011) 16:163–73. 10.1111/j.1440-1797.2010.01386.x 21272128

[102] Park SW Kim M Kim M D'Agati VD Thomas Lee H. Sphingosine kinase 1 protects against renal ischemia–reperfusion injury in mice by sphingosine-1-phosphate1 receptor activation. Kidney Int. (2011) 80:1315–27. 10.1038/ki.2011.281 21849969

[103] Lien YHH Yong KC Cho C Igarashi S Lai LW. S1P1-selective agonist, SEW2871, ameliorates ischemic acute renal failure. Kidney Int. (2006) 69:1601–8. 10.1038/sj.ki.5000360 16572108

[104] Bajwa A Rosin DL Chroscicki P Lee S Dondeti K Ye H. Sphingosine 1-phosphate receptor-1 enhances mitochondrial function and reduces cisplatin-induced tubule injury. J Am Soc Nephrol. (2015) 26:908–25. 10.1681/ASN.2013121351 25145931 PMC4378101

[105] Ham A Kim M Kim JY Brown KM Fruttiger M D'Agati VD. Selective deletion of the endothelial sphingosine-1-phosphate 1 receptor exacerbates kidney ischemia–reperfusion injury. Kidney Int. (2014) 85:807–23. 10.1038/ki.2013.345 24025642 PMC3952061

[106] Jo SK Bajwa A Ye H Vergis AL Awad AS Kharel Y. Divergent roles of sphingosine kinases in kidney ischemia-reperfusion injury. Kidney Int. (2009) 75:167–75. 10.1038/ki.2008.400 18971925 PMC2646633

[107] Won Park S Kim M Brown KM D'Agati VD Lee HT. Inhibition of sphingosine 1-phosphate receptor 2 protects against renal ischemia-reperfusion injury. J Am Soc Nephrol. (2012) 23:266–80. 10.1681/ASN.2011050503 22095950 PMC3269189

[108] Perry HM Huang L Ye H Liu C Sung S-SJ Lynch KR. Endothelial sphingosine 1-phosphate receptor-1 mediates protection and recovery from acute kidney injury. J Ame Soc Nephrol. (2016) 27:3383–93. 10.1681/ASN.2015080922 26961351 PMC5084889

[109] Li X Wu M Chen L Lu J Li G Fu L. A sphingosine-1-phosphate modulator ameliorates polycystic kidney disease in han:SPRD rats. Am J Nephrol. (2020) 51:1–10. 10.1159/000502855 31694015

[110] Huang K Liu W Lan T Xie X Peng J Huang J. Berberine reduces fibronectin expression by suppressing the S1P-S1P2 receptor pathway in experimental diabetic nephropathy models. PLoS ONE. (2012) 7:e43874. 10.1371/journal.pone.0043874 22937115 PMC3427312

[111] Lovric S Goncalves S Gee HY Oskouian B Srinivas H Choi WI. Mutations in sphingosine-1-phosphate lyase cause nephrosis with ichthyosis and adrenal insufficiency. J Clin Invest. (2017) 127:912–28. 10.1172/JCI89626 28165339 PMC5330730

[112] Janecke AR Xu R Steichen-Gersdorf E Waldegger S Entenmann A Giner T. Deficiency of the sphingosine-1-phosphate lyase SGPL1 is associated with congenital nephrotic syndrome and congenital adrenal calcifications. Hum Mutat. (2017) 38:365–72. 10.1002/humu.23192 28181337 PMC5384969

[113] Maharaj A Theodorou D Banerjee II Metherell LA Prasad R. A sphingosine-1-phosphate lyase mutation associated with congenital nephrotic syndrome and multiple endocrinopathy. Front Pediatr. (2020) 8:151. 10.3389/fped.2020.00151 32322566 PMC7156639

[114] Wang Z Sims CR Patil NK Gokden N Mayeux PR. Pharmacologic targeting of sphingosine-1-phosphate receptor 1 improves the renal microcirculation during sepsis in the mouse. J Pharmacol Exp Ther. (2015) 352:61–6. 10.1124/jpet.114.219394 25355645 PMC4279105

[115] Swendeman SL Xiong Y Cantalupo A Yuan H Burg N Hisano Y. An engineered S1P chaperone attenuates hypertension and ischemic injury. Sci Sig. (2017) 10:eaal2722. 10.1126/scisignal.aal2722 28811382 PMC5680089

